# On the Diagnosis of Malignant Disease of the Body of the Uterus, with Notes of a Case Treated Twice by Curettage, and Finally by Hysterectomy
1Read at the Meeting of the Bristol Medico-Chirurgical Society, March 11th, 1903.


**Published:** 1903-06

**Authors:** Ernest W. Hey Groves


### ON THE DIAGNOSIS OF MALIGNANT DISEASE OF THE BODY OF THE UTERUS, WITH NOTES OF A CASE TREATED TWICE BY CURETTAGE, AND FINALLY BY HYSTERECTOMY.[1]

BY

Ernest W. Hey Groves, M.D., B.Sc. (Lond.).

---

Cases of malignant disease of the body of the uterus are undoubtedly rare, forming, according to Schrœder, only 3·4 per cent. of all the cases of malignant affections of the uterus. And when a leading English authority speaks of only having seen five cases[2], it is clear that in the experience of any one practitioner the occurrence of such a case will be so uncommon as to compel him to rely on the experience of others in its diagnosis. The great importance of early diagnosis in this disease is only equalled by its difficulty; and as the following case illustrates this difficulty so clearly, I bring it forward in the hope that it may afford a good subject for discussion. And I must admit from the outset that I am fully prepared to have the diagnosis of malignancy disputed, and the radical treatment of hysterectomy, which follows from that diagnosis, called in question.

The patient was a maiden lady, aged 47, who, when I saw her in 1901 first, had the following history: No previous illness. Catamenia used to recur every 28 days, last six days without much loss or pain.

1892. Periods began to be prolonged to 7–9 days, and she consulted a doctor, who said she had a tumour.

1893. She once had "a flooding" during a menstrual period, when she passed "large clots."

For six years the lengthened periods continued without any further "flooding" or other feature until 1899, when each period was followed by several days of severe pain, which confined her to bed. The pain was in the left side of the genital organs, and shooting down the left leg. The next year, 1900, she had four to six days' blood-stained discharge after each period ceased, as

[1] Read at the Meeting of the Bristol Medico-Chirurgical Society, March 11th, 1903.

[2] Sinclair, Clifford Allbutt and Playfair's *System of Gynæcology*, 1896, p. 715.

well as the great pain. This condition continued to get worse in spite of rest, drugs and douches, until, in 1901, she was bleeding for fourteen days out of every twenty-eight, and was reduced by pain and anæmia to absolute invalidism. In November, 1901, she was seen by a London gynæcologist, who removed a "large mucous polypus from the uterine cavity and curetted"; but bleeding continued after this operation for fourteen days. During the next five months all the previous symptoms reappeared, so that a week's copious bleeding was followed by nearly a week of thin sanious discharge, which was never offensive; the pain in the left labium and left leg was worse than ever, and during these months she spent nearly all her time between the bed and sofa.

On examination, the uterus could be felt to be somewhat enlarged, hard and tender, but fully movable: on its posterior surface a hard nodule was felt; the sound passed with great pain, $3\frac{1}{2}$ inches with concavity backwards, and produced copious bleeding. An exploratory operation was undertaken on April 9th with a view to discovering the condition of the uterine cavity. In the lithotomy position, under anæsthesia, the cervix was dilated by Hegar's dilators until the finger could be introduced. The cavity was irregular, being lined by thickened mucous membrane, which projected in ridges and mounds; but no discrete tumour or polypus was felt: every manipulation caused very free bleeding. The sharp curette was used, and could be felt to cut deeply into the thick mucous membrane, especially in the region of the fundus, and more than an ounce (fluid measure) of coarsely granular material removed. The uterine wall then sounded harsh and grating, but smooth, under the curette.

All this seemed perfectly typical of endometritis fungosa, and, with confidence in this diagnosis, I forwarded a specimen from the curettage to an eminent pathologist who, to my great surprise, returned a report of malignancy. I felt such difficulty in accepting this, however, that I sent a second specimen to another well-known pathologist who, without any hesitation, declared it to be columnar-celled carcinoma, his description running as follows: "Both these fragments from the uterus are affected with columnar-celled carcinoma of the villous type. The surface exhibits many papillary outgrowths, and here and there cysts are formed, containing similar papillomata. The evidence of malignancy is found in the processes of epithelial cells which are invading the muscular substance of the uterus. Many of the tubules are filled with mucoid secretion." It seemed folly to doubt or delay after this, and accordingly three days later the uterus was removed by vaginal hysterectomy. The operation was rendered difficult by the smallness of the vagina, which was enlarged by cutting right through the perineum to the sphincter ani. The uterus could not be extracted without almost cutting it in two. Three pair of forceps were left on the broad ligaments, and the ligatures left long, the vagina being sewn together with one stitch at the pelvic floor and with four at the perineum.

The same afternoon I received a second report from the first pathologist I had consulted, saying that after further sections had been cut "they present some difficulties, but, on the whole, I am inclined to think that the growth is non-malignant. Some appearances, however, are not unlike adeno-carcinoma, and therefore I would advise a further curetting in three months' time."

For the next few days whilst the patient's life trembled in the balance this report made me very unhappy, but I am glad to say she made an uneventful recovery and is now quite well and active.

The uterus was submitted to both authorities. The first now holds by his later opinion of non-malignancy. The second writes: "This uterus was so hard and distorted on arrival that at first sight there appeared to be no growth in its cavity. The uterus, as a whole, is hypertrophied, which is partly due to the presence of many small fibroids in the wall. The lining of the uterus has a rusty colour from old blood staining. At the fundus there are several ragged areas in the endometrium, but little or no projecting growth. The section has been taken from one such area, and at right angles to the surface. It exhibits tubular processes of columnar-celled carcinoma deeply infiltrating the muscular tissue. Some of the tubules form small cysts containing villous ingrowths, and in other places the cells are undergoing mucoid degeneration. Hence the condition exactly corresponds with that previously found in the curettings."

I would add to this description of the uterus the remark that the bulk of the new growth proper has been scraped out by the curette, which was applied only ten days before the organ was removed; and if the growth had been left *in situ* the organ would not have exhibited its present innocent appearance.

I may now very briefly call your attention to the chief symptoms of carcinoma of the body of the uterus in their bearing upon the diagnosis between malignant and benign disease.

*Etiology.*—The patients are older women than is usual in carcinoma of the cervix. The age is seldom or never below 45 years, and is usually much greater than this, 55 being a common average. Thus more than half the cases occur long after the climacteric. This would serve to distinguish them from the majority of cases of fibroids, and also from cases in which septic endometritis has complicated incomplete abortion. The patients are usually nulliparous, and often belong to the better social classes.

*Bleeding* is always the first and most prominent symptom. Insidious in its onset and indefinite in its characters, it is long concealed by the patient, and even when described to the medical man is too likely to be made light of, especially as it is exceptional for any tumour to be found on examination. It has the character of menorrhagia in all ante-climacteric and in most post-climacteric cases.

*Pain*, however, soon supervenes and accompanies the bleeding, being, in fact, symptomatic dysmenorrhœa. Professor Sinclair suggests that this is due to the effort on the part of the uterus to expel the diseased endometrium as though it were a foreign body. But however this may be, the pain is an early symptom, is paroxysmal, and usually absent between the attacks of hemorrhage. It is very different from the agony of cancer of the cervix, which comes on late in the disease, and when once established is hardly ever absent, being due as it is to a malignant infiltration of the connective tissue and nerves of the pelvis.

*Discharge.*—When the disease is well established the intervals between the bleeding are occupied by a thin sanious flux, which is not particularly offensive, and it is only in advanced cases that this becomes fœtid or purulent.

*General Condition and Course of Disease.*—Cachexia and emaciation are long delayed, the patient's condition being that of anæmia without any loss of flesh for several years. It seldom proves fatal in less than five years from the onset of the symptoms, and may extend over eight, nine, or even ten years.

*Local Conditions of the Disease.*—The uterus is somewhat enlarged and hard, but freely movable, until the peritonitis which closes the case begins to tie it down by adhesions. The cervix is unchanged, or it may be thinned out by the partial protrusion of a polypoid growth, and its mucous membrane often has an unhealthy dark-blue appearance. There is no invasion of the connective tissue in the broad ligaments or round the rectum; in fact, there is nothing of diagnostic importance that can be discovered by vaginal or bimanual palpation. Metastatic growths appear more slowly in this than in any other form of visceral malignant disease; when they do occur it is in the lumbar and not the pelvic glands.

All these features of carcinoma of the body of the uterus offer a marked contrast to those of cancer of the cervix, but these features are due to the situation rather than to the nature of the growth, and are shared by other diseases of the endometrium and body of the uterus.

There are at least four diseases which produce some or all of the symptoms of carcinoma of the body of the uterus, and of these two are readily dismissed. They are incomplete abortion, fibroids, mucous polypus, and certain mis-named forms of endometritis.

*Incomplete Abortion with Septic Inflammation of the Uterus.*—In this condition the history will be a short one, measured by months and not years. The symptoms begin abruptly with a so-called "flooding" preceded probably by amenorrhœa. The uterus, although enlarged, is soft, and the os patulous; bleeding is copious and irregular, and the discharge often very foul with shreds and clots; pain is not conspicuous, and is of a dull aching character. On dilating the cervix the nature of the uterine contents is readily recognised to be that of fœtal membranes or placenta, or it may be that a fibrinous polypus has been moulded on the disorganised shreds of these products of conception.

*Fibroids.*—In the majority of cases a slowly increasing menorrhagia with delayed menopause is associated with irregular or great uterine enlargement. On exploring the uterine cavity and curetting [1] a small quantity of shreddy material is removed amounting to scarcely a drachm, and the uterine wall is felt to be hard and smooth. A fibrous polypus may be found in the uterine cavity or may be already extracted through the cervix. The firmness of this and its dense white appearance on section leave no doubt as to its nature. Sometimes, however, a fibroid may have remained latent and the menopause established, when (it may be years afterwards) the fibroid sloughs, producing bleeding, copious and very offensive discharge, with some pain. If necrosis has advanced so far as to

[1] Howard Kelly, *Operative Gynecology*, vol. i., 1898, p. 491.

destroy the bulk of the fibroid before the case comes under observation, the abundant shreddy material scraped out from the uterus will have a fibrous character, as seen by the microscope, which could not be mistaken for anything else.

*Mucous polypus*, or, better, simple polypoid adenoma, causes copious bleeding with profound anæmia if left untreated; but there is a marked absence of pain such as occurs early in the history of malignant disease of the body of the uterus. If the polypus is not extruding from the cervix, the latter must be dilated, and then a soft growth, varying in size from that of a lentil up to that of a walnut, is found slenderly attached to the wall of the uterus. It is readily twisted off, and after the uterus has been curetted the symptoms usually cease, and this is notably the case when the polypus is attached in the neighbourhood of the cervix. In women between 45 and 50 this simple operation is enough to establish the menopause, and generally uterine bleeding ceases from that day onwards, or only one or two scanty periods occur before permanent amenorrhœa is established. But in a few cases this desirable sequel to the removal of the polypoid adenoma does not occur, and, as in the instance I have related, the bleeding and pain are rapidly re-established, and the case must be regarded as one of "hypertrophic endometritis" or malignant disease. The histological nature of the mucous polypus will not guide us much, showing simply the structure of an adenoma; therefore in a case of a mucous polypus, whatever the pathological report, it would be wise to give a guarded prognosis, and not to decide the question of recurrence in a benign or malignant form until several months have elapsed.

*Hypertrophic Endometritis, or, better, Adenoma of the Uterus*, includes a group of cases which contains all pathological gradations between a simple mucous polypus and fully-developed adeno-carcinoma. Great confusion has been introduced into this subject by the multiplication of names, most of which are fundamentally incorrect in implying a primary inflammatory condition; thus, endometritis hypertrophica, E. hyperplastica, E. villosa, and E. fungosa, are all names for a new growth of the uterine mucous membrane; but although all text-books now point this out, yet the old term endometritis is still used to designate all that group of cases where the mucous membrane is thickened and the glandular elements greatly increased in number and complexity without showing malignant characters. Calling, then, all these conditions simple adenoma of the uterus, let us consider what are their symptoms and pathology.

The symptoms are the same as those of the mucous polypus, as might be expected from essentially the same disease. Bleeding, with the consequent symptoms of anæmia, sums up the patient's complaint. There may be some intermenstrual discharge of thin blood-stained inodorous fluid, but the main bleeding is a prolonged successive monthly loss. Pain is conspicuously absent, and this affords the most striking difference from malignant disease. The patients are of exactly the same age as those subject to cancer of the body; they are often nullipara who have passed the climacteric. For this reason the adenoma has often been called endometritis senilis; but this name ought to be retained for a very rare condition, which is essentially one of atrophy, in which the shreddy *débris* of the mucous membrane contains hardly any glandular elements, and which cannot therefore be mistaken either for adenoma or cancer.

On examining the uterine cavity in adenoma the mucous membrane is thick and soft, and bleeds profusely on manipulation; it is thrown into more or less marked folds, tags, villous projections or polypi, which can be readily removed with the curette. Under the microscope sections of this tissue show the same structure as that of the mucous polypus, with perhaps greater elaboration. There is an increase in both interstitial and glandular elements of the mucous membrane, although the first predominates in one case and the second in another. The glands are enlarged and tortuous: some are dilated to form cysts, and many show mucoid or myxomatous changes. So far there is nothing to suggest malignant disease, but unfortunately two further features may be presented which are supposed to be typical of cancer. The one is endothelial proliferation, producing a blocking of the glands, and the other is an invasion of the muscular wall of the uterus by the glandular elements. The first of these points is the common criterion of carcinoma, and yet it not only occurs in simple adenoma, but is actually described as the normal condition in the deeper portions of the glands by Symington in Quain's *Anatomy*.[1] There is no doubt a great difference between an established malignant growth composed largely of solid epithelial tubules and a simple adenoma in which all the glands are well formed and with a good lumen; but still, in a doubtful case, it is hardly possible to rely for decision on a feature which may actually be found in the normal uterus.

The invasion of the muscular tissue by glandular elements is said to occur, and is figured by many writers, *e.g.*, Cornil,[2] Cullen,[3] and Eden,[4] in cases of simple adenoma or hypertrophic endometritis. It would be interesting, however, to know the after-history of these cases before admitting their benign nature. For instance, at the Obstetrical Society, in 1900, Dr. Eden showed almost exactly similar microscopic sections to those of the case I have just described. He called it a fibro-adenoma, and figures the glandular processes deeply invading the muscular tissue; but the woman died of pulmonary embolism, and the benign nature of the growth was a mere conjecture.

In their clinical course, too, many cases of adenoma resemble cancer in their great tendency to recurrence. For example, Haultain[5] describes a case in which "Five years ago, for the fourth time within eighteen months, I removed from a patient aged 59, still alive and healthy, a large number of intra-uterine adenomas." And he believes so much in the simple nature of such conditions that he goes so far as to remark: "As these neoplasms have been known to be the forerunners of malignant disease, and also in some cases to recur locally, a chance is given to those smitten with the hysterectomy furor to remove the uterus." Sir John Williams,[6] on the other hand (in common

1 Clifford Allbutt and Playfair, *op. cit.*, p. 714.

2 Cornil, *Leçons sur l'Anatomie pathologique des Metrites*, Paris, 1889.

3 Cullen, *Cancer of the Uterus*, 1900.

4 Eden, *Tr. Obst. Soc. Lond.*, 1900, xlii. 2.

5 Haultain in Clifford Allbutt and Playfair, *op. cit.*, p. 609.

6 *Cancer of the Uterus*, 1888.

with many continental authors), expresses the view, in his Harveian lectures on *Cancer of the Uterus*, that all cases of adenoma occurring at or after the menopause are malignant and should be dealt with as such.

Two facts, however, seem to be established amid all this uncertainty. The first is that in its early stages adeno-carcinoma of the body of the uterus cannot be distinguished by microscopical characters alone from adenoma, or the so-called endometritis hypertrophica; and the second is that all these glandular new growths of the endometrium run one of three clinical courses; either they never recur after removal with curetting, or they recur a number of times without fresh evidence of malignancy, and are finally cured by curetting, or they recur in a more pronounced malignant form, and end, if not treated radically, as frank malignant disease.

The practical deduction from these facts seems to be that every case in which excessive bleeding occurs at or after the menopause, and in which the curette removes a glandular polypus or a quantity of richly glandular new growth, should be regarded with suspicion, and that every recurrence of such growth and the rapidity of the recurrence should heighten this suspicion, and that if the disease be not cured within a reasonable time, *i.e.*, a year or eighteen months, the uterus ought to be removed without further delay. And I may here remark that few patients are sufficiently philosophical to submit to a curetting under anæsthesia every three or four months whilst this result is being arrived at, in which case it would be better to remove the uterus if the symptoms recur after the second curetting than to allow the woman to trust to the chances of a purely expectant treatment.

In the case I have described there are, besides the pathological report, two strong indications of malignancy. In the first place, the symptoms recurred at once after curetting without any free interval at all; and, second, the large quantity of new growth formed in the short interval of four months.

The case was complicated by the fibroids in the wall of the uterus, which no doubt accounts for the ten years' moderate menorrhagia which preceded the more acute symptoms.

The excellent results obtained by vaginal hysterectomy in this disease fully compensate for the difficulty in its diagnosis. Thus Wilson, in the *Journal of Obstetrics and Gynæcology*,[1] shows that the proportion of lasting (*i.e.*, three years) cures after hysterectomy for cancer of the body of the uterus is 75 per cent., whereas the corresponding figure for cases of cancer of the cervix is only 25 per cent. This is due to the great thickness of the uterine wall which limits the growth, to the resulting fact that invasion of surrounding organs does not occur, and to the very late development of metastasis.

---

### MISSING LINKS IN JOINT DISEASE.

[1] Wilson, *J. Obst. and Gynæc.*, 1902, p. 542.

